# Supplementary material for: The Nucleome Data Bank: web-based resources to simulate and analyze the three-dimensional genome
Source: Nucleic Acids Res. 2020 Oct 6;49(D1):D172–82. doi: 10.1093/nar/gkaa818 (PMC7778995; doi:10.1093/nar/gkaa818)
Supplement: gkaa818_Supplemental_File [file gkaa818_supplemental_file.pdf]

Supporting Information for The Nucleome Data Bank: Web-based Resources to Simulate and Analyze the Three-Dimensional Genome.

Vinícius G. Contessoto,[*,†,‡] Ryan R. Cheng,[†] Arya Hajitaheri,[†,¶] Esteban Dodero-Rojas,[†,§] Matheus Faria Mello,[†,‖] Erez Lieberman-Eiden,[†,⊥] Peter G. Wolynes,[†,#,@,△] Michele Di Pierro,[*,†,∇] and José N. Onuchic[*,†,#,@,△]

†*Center for Theoretical Biological Physics, Rice University, Houston, TX, USA*

‡*Brazilian Biorenewables National Laboratory - LNBR, Brazilian Center for Research in Energy and Materials - CNPEM, Campinas, SP, Brazil*

¶*Department of Computer Science, University of Houston, Houston, TX, USA*

§*Theoretical and Computational Physics Laboratory, University of Costa Rica, San José, Costa Rica*

‖*Chemical Engineering Department, Military Institute of Engineering, Rio de Janeiro, RJ, Brazil*

⊥*The Center for Genome Architecture, Department of Molecular and Human Genetics, Baylor College of Medicine, Houston, TX , USA*

#*Department of Physics & Astronomy, Rice University, Houston, TX, USA*

@*Department of Chemistry, Rice University, Houston, TX, USA*

△*Department of Biosciences, Rice University, Houston, TX, USA*

∇*Department of Physics, Northeastern University, Boston, MA, USA*

E-mail: vinicius.contessoto@rice.edu; m.dipierro@northeastern.edu; jonuchic@rice.edu

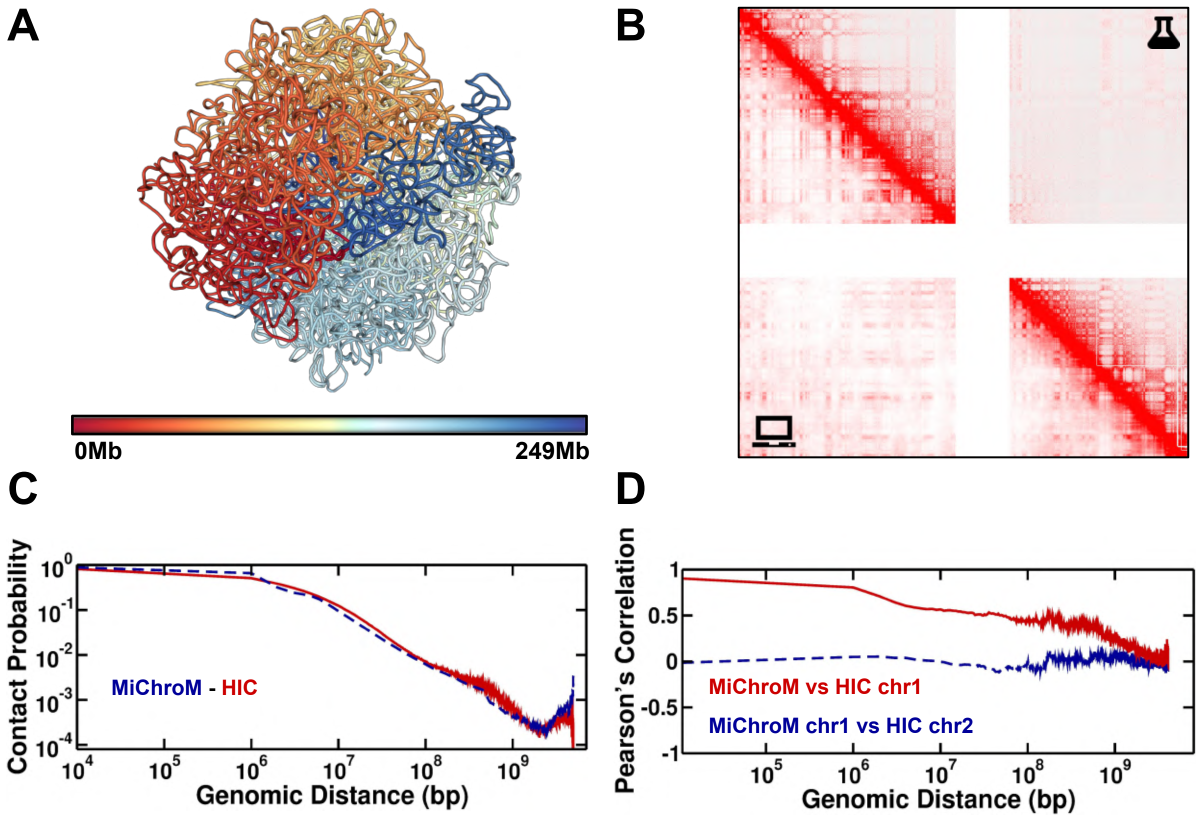

Figure S1: The structural ensemble of chromosome 1, cell line A549, generated by simulations using the MiChroM+MEGABASE pipeline. A - Three-dimensional representative structure colored by index, red to blue. B - Comparison between Hi-C maps obtained from wet-lab experiments (top) and *in silico* (bottom). The Hi-C maps predicted from simulations have been sampled from an ensemble of 500 thousand structures. C - Contact probability as a function of the genomic distance. The solid red line is the curve extracted from the experimental data. The dashed blue line is the data obtained from the *in silico* HiC. D - Pearson's correlation between experimental and simulated Hi-C maps of chromosome 1 as a function of the genomic distance, shown by the solid red line. As a term of comparison the dashed blue line shows the correlation between Hi-C maps of different chromosomes.

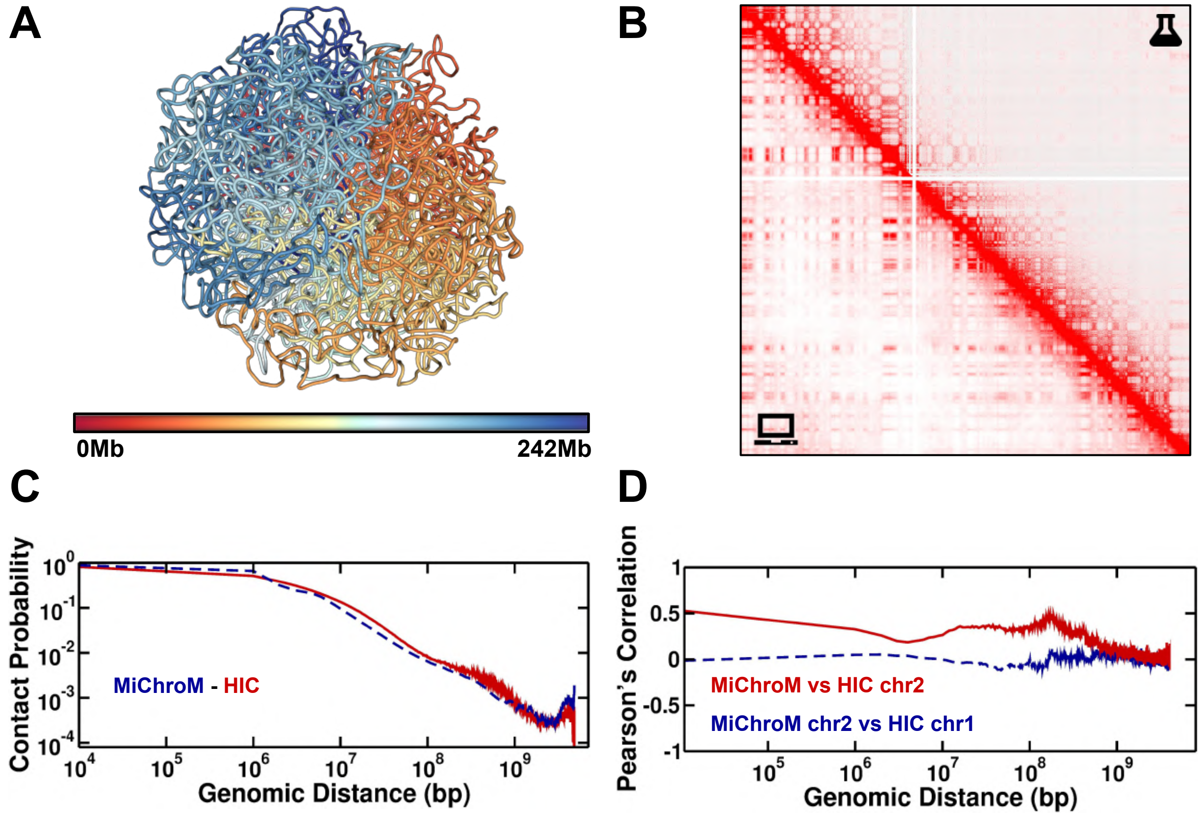

Figure S2: The structural ensemble of chromosome 2, cell line A549, generated by simulations using the MiChroM+MEGABASE pipeline. A - Three-dimensional representative structure colored by index, red to blue. B - Comparison between Hi-C maps obtained from wet-lab experiments (top) and *in silico* (bottom). The Hi-C maps predicted from simulations have been sampled from an ensemble of 500 thousand structures. C - Contact probability as a function of the genomic distance. The solid red line is the curve extracted from the experimental data. The dashed blue line is the data obtained from the *in silico* HiC. D - Pearson's correlation between experimental and simulated Hi-C maps of chromosome 2 as a function of the genomic distance, shown by the solid red line. As a term of comparison the dashed blue line shows the correlation between Hi-C maps of different chromosomes.

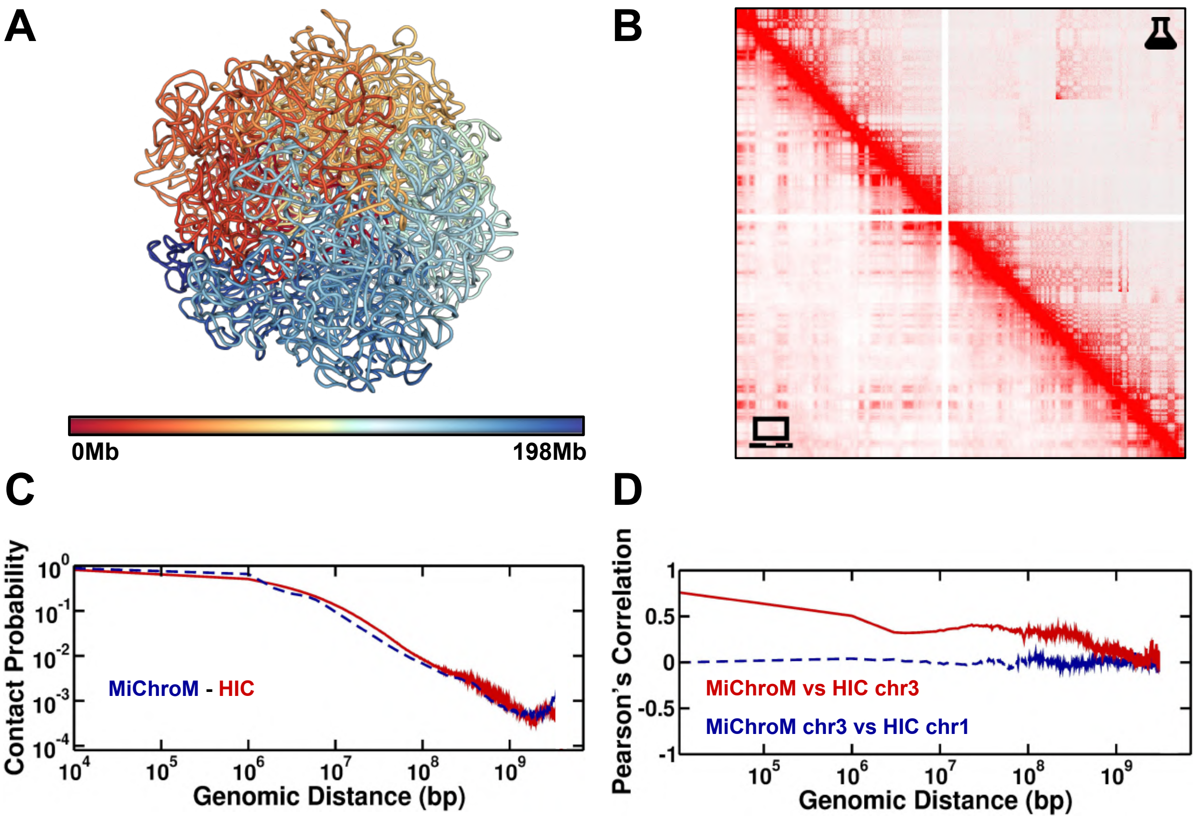

Figure S3: The structural ensemble of chromosome 3, cell line A549, generated by simulations using the MiChroM+MEGABASE pipeline. A - Three-dimensional representative structure colored by index, red to blue. B - Comparison between Hi-C maps obtained from wet-lab experiments (top) and *in silico* (bottom). The Hi-C maps predicted from simulations have been sampled from an ensemble of 500 thousand structures. C - Contact probability as a function of the genomic distance. The solid red line is the curve extracted from the experimental data. The dashed blue line is the data obtained from the *in silico* HiC. D - Pearson's correlation between experimental and simulated Hi-C maps of chromosome 3 as a function of the genomic distance, shown by the solid red line. As a term of comparison the dashed blue line shows the correlation between Hi-C maps of different chromosomes.

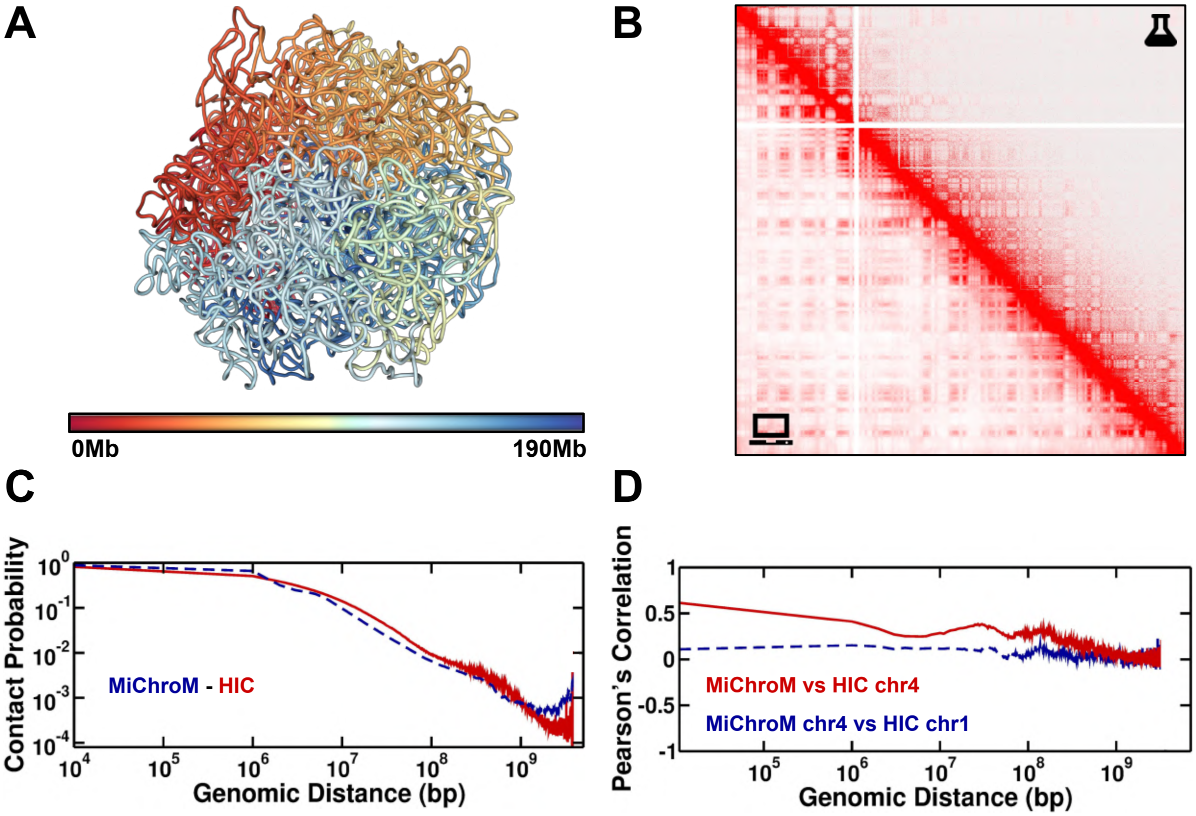

Figure S4: The structural ensemble of chromosome 4, cell line A549, generated by simulations using the MiChroM+MEGABASE pipeline. A - Three-dimensional representative structure colored by index, red to blue. B - Comparison between Hi-C maps obtained from wet-lab experiments (top) and *in silico* (bottom). The Hi-C maps predicted from simulations have been sampled from an ensemble of 500 thousand structures. C - Contact probability as a function of the genomic distance. The solid red line is the curve extracted from the experimental data. The dashed blue line is the data obtained from the *in silico* HiC. D - Pearson's correlation between experimental and simulated Hi-C maps of chromosome 4 as a function of the genomic distance, shown by the solid red line. As a term of comparison the dashed blue line shows the correlation between Hi-C maps of different chromosomes.

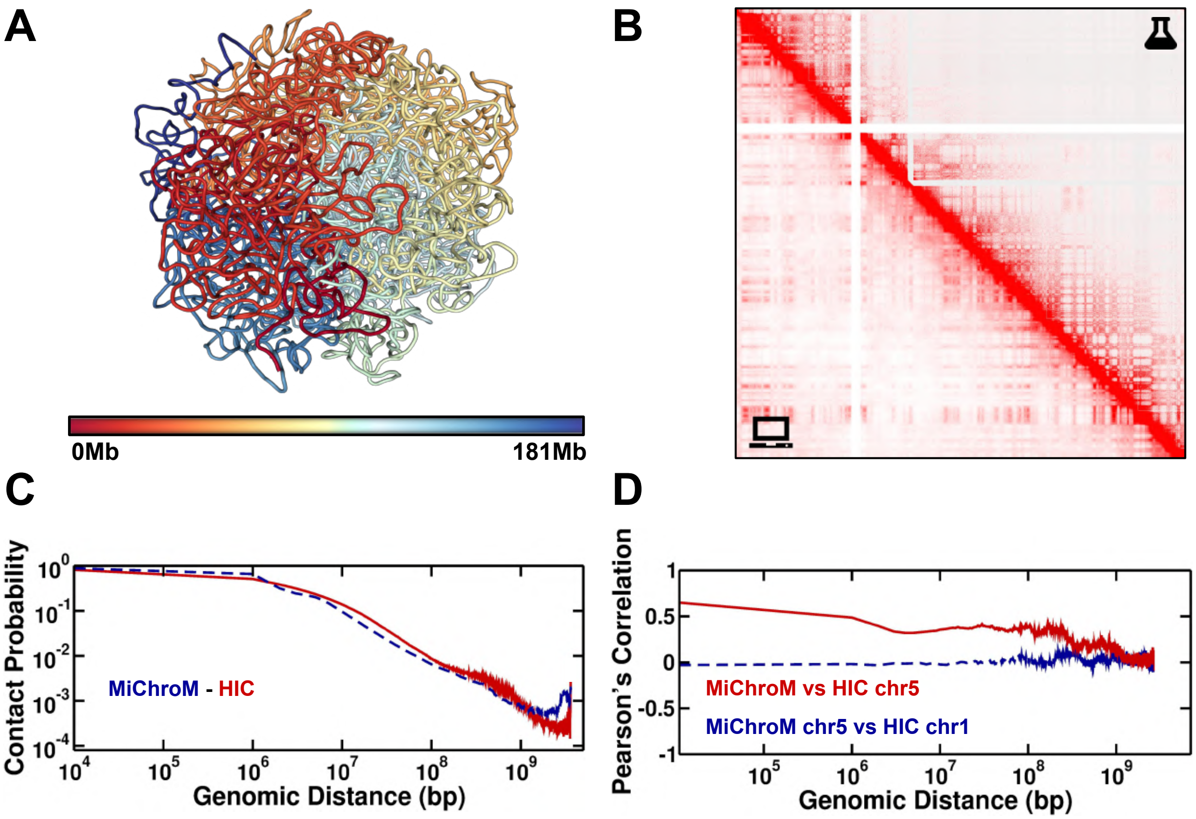

Figure S5: The structural ensemble of chromosome 5, cell line A549, generated by simulations using the MiChroM+MEGABASE pipeline. A - Three-dimensional representative structure colored by index, red to blue. B - Comparison between Hi-C maps obtained from wet-lab experiments (top) and *in silico* (bottom). The Hi-C maps predicted from simulations have been sampled from an ensemble of 500 thousand structures. C - Contact probability as a function of the genomic distance. The solid red line is the curve extracted from the experimental data. The dashed blue line is the data obtained from the *in silico* HiC. D - Pearson's correlation between experimental and simulated Hi-C maps of chromosome 5 as a function of the genomic distance, shown by the solid red line. As a term of comparison the dashed blue line shows the correlation between Hi-C maps of different chromosomes.

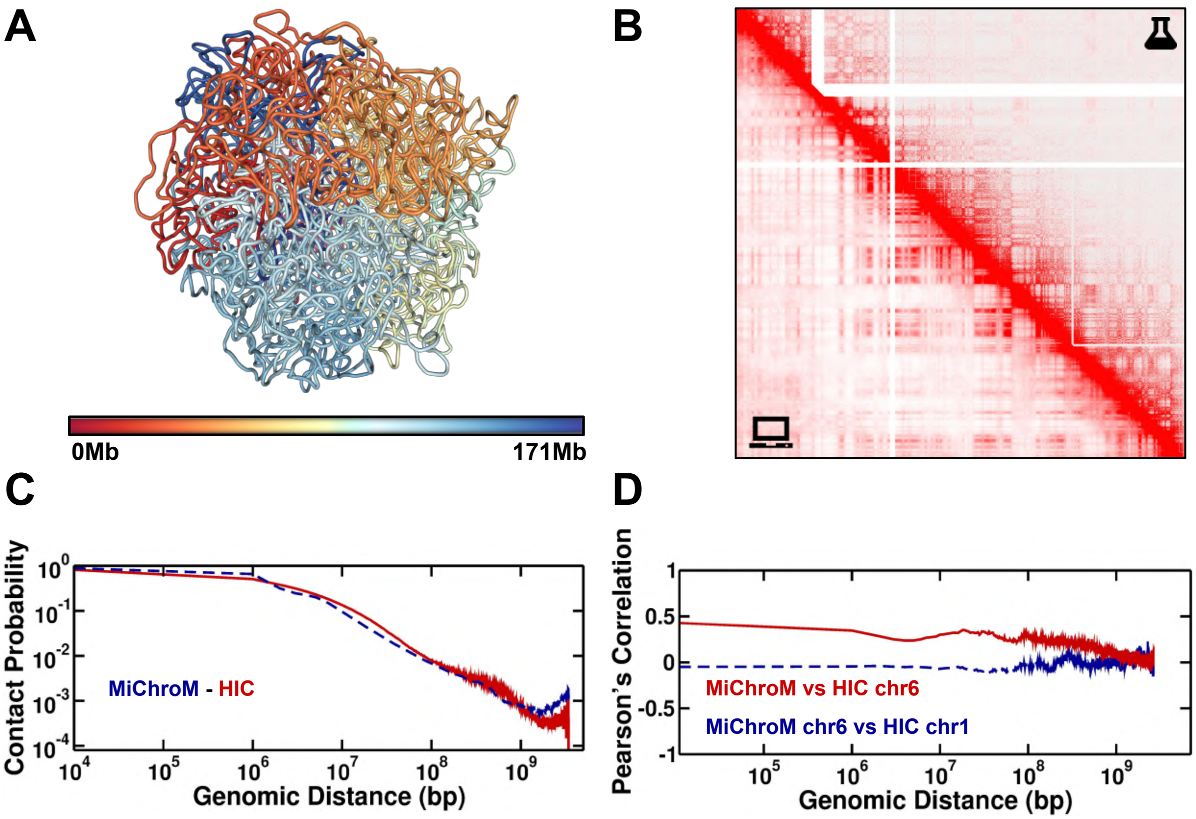

Figure S6: The structural ensemble of chromosome 6, cell line A549, generated by simulations using the MiChroM+MEGABASE pipeline. A - Three-dimensional representative structure colored by index, red to blue. B - Comparison between Hi-C maps obtained from wet-lab experiments (top) and *in silico* (bottom). The Hi-C maps predicted from simulations have been sampled from an ensemble of 500 thousand structures. C - Contact probability as a function of the genomic distance. The solid red line is the curve extracted from the experimental data. The dashed blue line is the data obtained from the *in silico* HiC. D - Pearson's correlation between experimental and simulated Hi-C maps of chromosome 6 as a function of the genomic distance, shown by the solid red line. As a term of comparison the dashed blue line shows the correlation between Hi-C maps of different chromosomes.

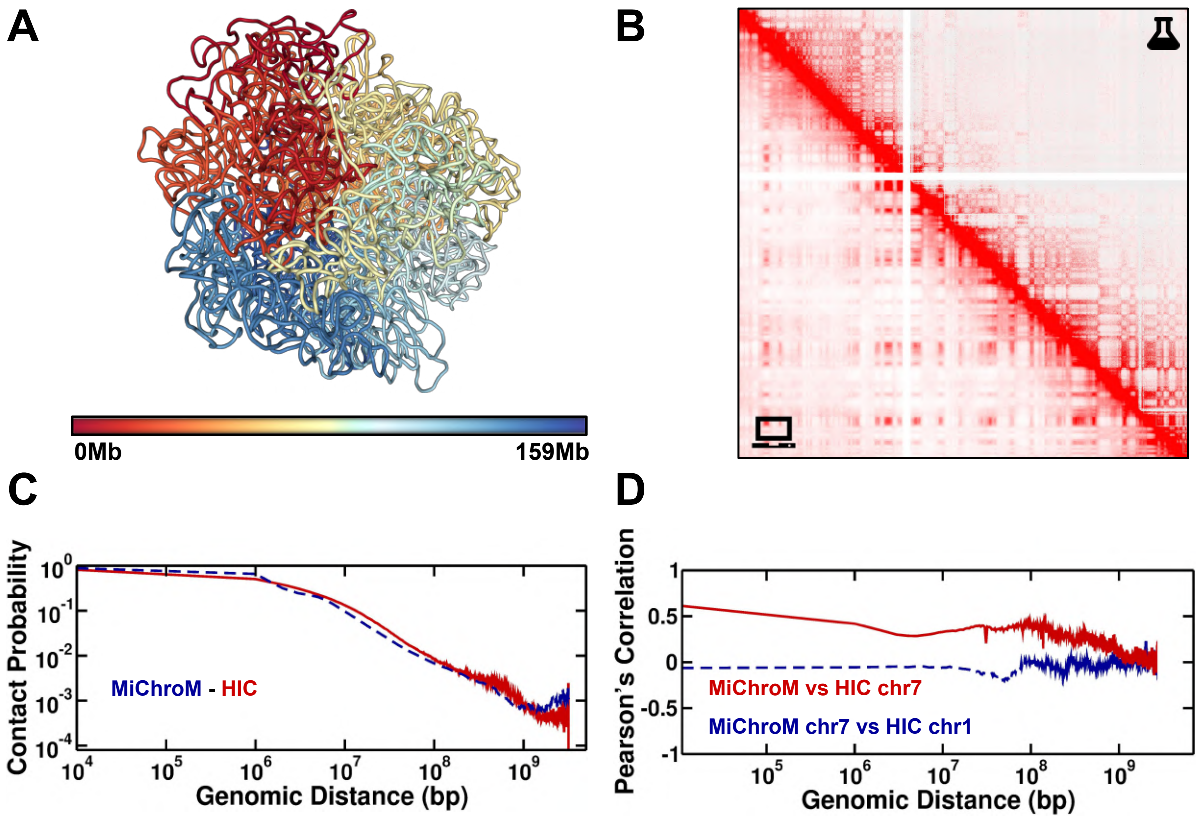

Figure S7: The structural ensemble of chromosome 7, cell line A549, generated by simulations using the MiChroM+MEGABASE pipeline. A - Three-dimensional representative structure colored by index, red to blue. B - Comparison between Hi-C maps obtained from wet-lab experiments (top) and *in silico* (bottom). The Hi-C maps predicted from simulations have been sampled from an ensemble of 500 thousand structures. C - Contact probability as a function of the genomic distance. The solid red line is the curve extracted from the experimental data. The dashed blue line is the data obtained from the *in silico* HiC. D - Pearson's correlation between experimental and simulated Hi-C maps of chromosome 7 as a function of the genomic distance, shown by the solid red line. As a term of comparison the dashed blue line shows the correlation between Hi-C maps of different chromosomes.

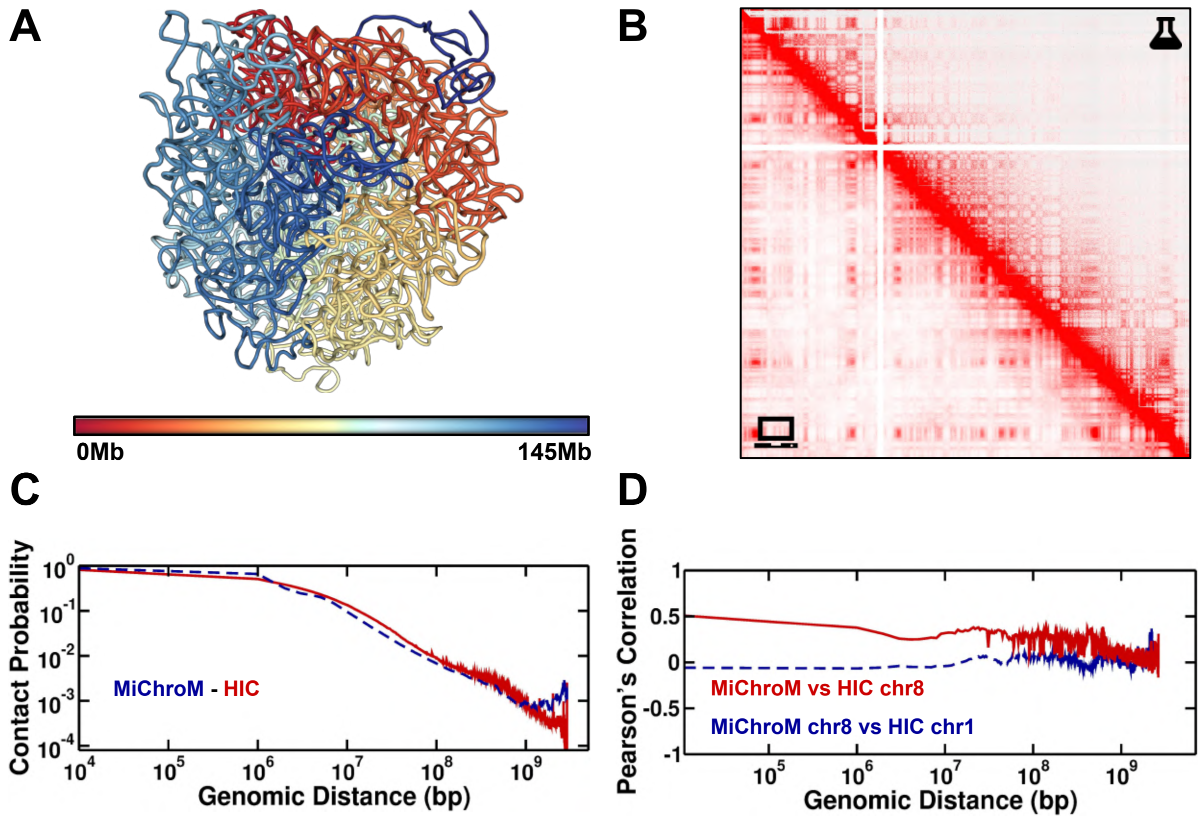

Figure S8: The structural ensemble of chromosome 8, cell line A549, generated by simulations using the MiChroM+MEGABASE pipeline. A - Three-dimensional representative structure colored by index, red to blue. B - Comparison between Hi-C maps obtained from wet-lab experiments (top) and *in silico* (bottom). The Hi-C maps predicted from simulations have been sampled from an ensemble of 500 thousand structures. C - Contact probability as a function of the genomic distance. The solid red line is the curve extracted from the experimental data. The dashed blue line is the data obtained from the *in silico* HiC. D - Pearson's correlation between experimental and simulated Hi-C maps of chromosome 8 as a function of the genomic distance, shown by the solid red line. As a term of comparison the dashed blue line shows the correlation between Hi-C maps of different chromosomes.

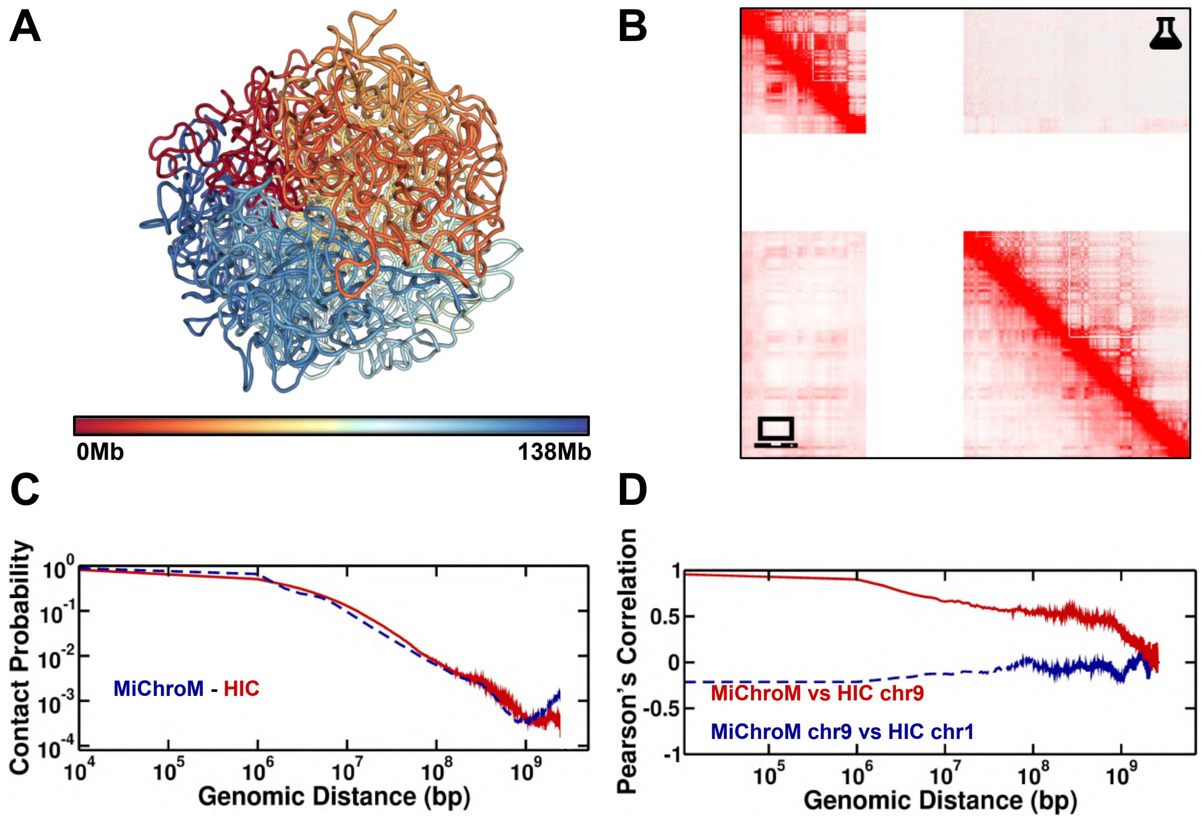

Figure S9: The structural ensemble of chromosome 9, cell line A549, generated by simulations using the MiChroM+MEGABASE pipeline. A - Three-dimensional representative structure colored by index, red to blue. B - Comparison between Hi-C maps obtained from wet-lab experiments (top) and *in silico* (bottom). The Hi-C maps predicted from simulations have been sampled from an ensemble of 500 thousand structures. C - Contact probability as a function of the genomic distance. The solid red line is the curve extracted from the experimental data. The dashed blue line is the data obtained from the *in silico* HiC. D - Pearson's correlation between experimental and simulated Hi-C maps of chromosome 9 as a function of the genomic distance, shown by the solid red line. As a term of comparison the dashed blue line shows the correlation between Hi-C maps of different chromosomes.

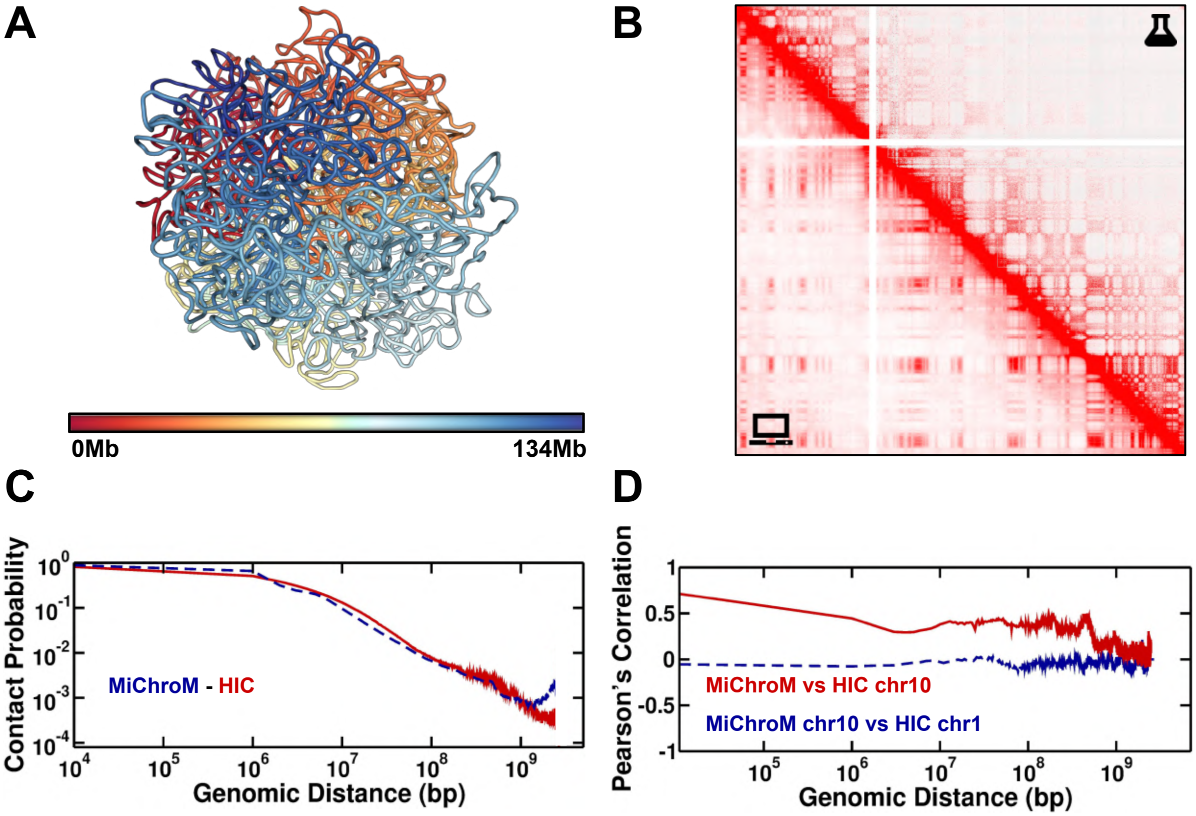

Figure S10: The structural ensemble of chromosome 10, cell line A549, generated by simulations using the MiChroM+MEGABASE pipeline. A - Three-dimensional representative structure colored by index, red to blue. B - Comparison between Hi-C maps obtained from wet-lab experiments (top) and *in silico* (bottom). The Hi-C maps predicted from simulations have been sampled from an ensemble of 500 thousand structures. C - Contact probability as a function of the genomic distance. The solid red line is the curve extracted from the experimental data. The dashed blue line is the data obtained from the *in silico* HiC. D - Pearson's correlation between experimental and simulated Hi-C maps of chromosome 10 as a function of the genomic distance, shown by the solid red line. As a term of comparison the dashed blue line shows the correlation between Hi-C maps of different chromosomes.

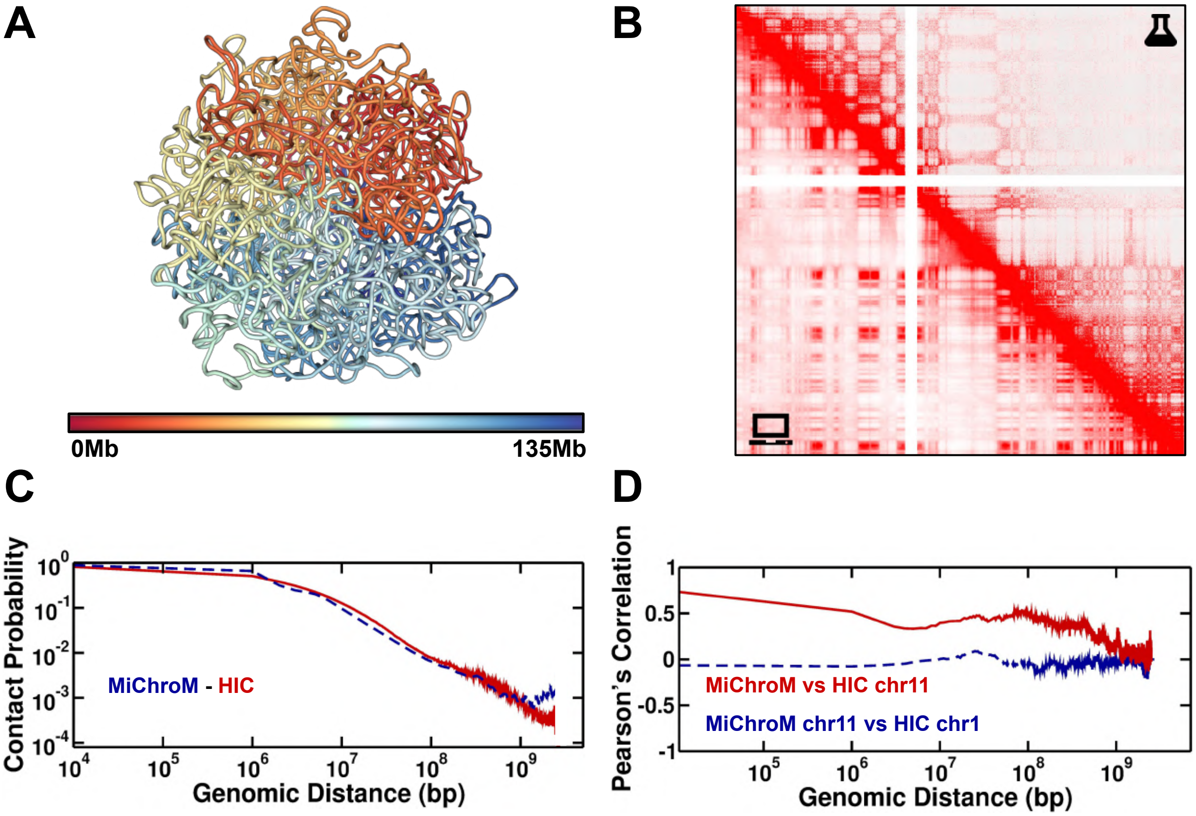

Figure S11: The structural ensemble of chromosome 11, cell line A549, generated by simulations using the MiChroM+MEGABASE pipeline. A - Three-dimensional representative structure colored by index, red to blue. B - Comparison between Hi-C maps obtained from wet-lab experiments (top) and *in silico* (bottom). The Hi-C maps predicted from simulations have been sampled from an ensemble of 500 thousand structures. C - Contact probability as a function of the genomic distance. The solid red line is the curve extracted from the experimental data. The dashed blue line is the data obtained from the *in silico* HiC. D - Pearson's correlation between experimental and simulated Hi-C maps of chromosome 11 as a function of the genomic distance, shown by the solid red line. As a term of comparison the dashed blue line shows the correlation between Hi-C maps of different chromosomes.

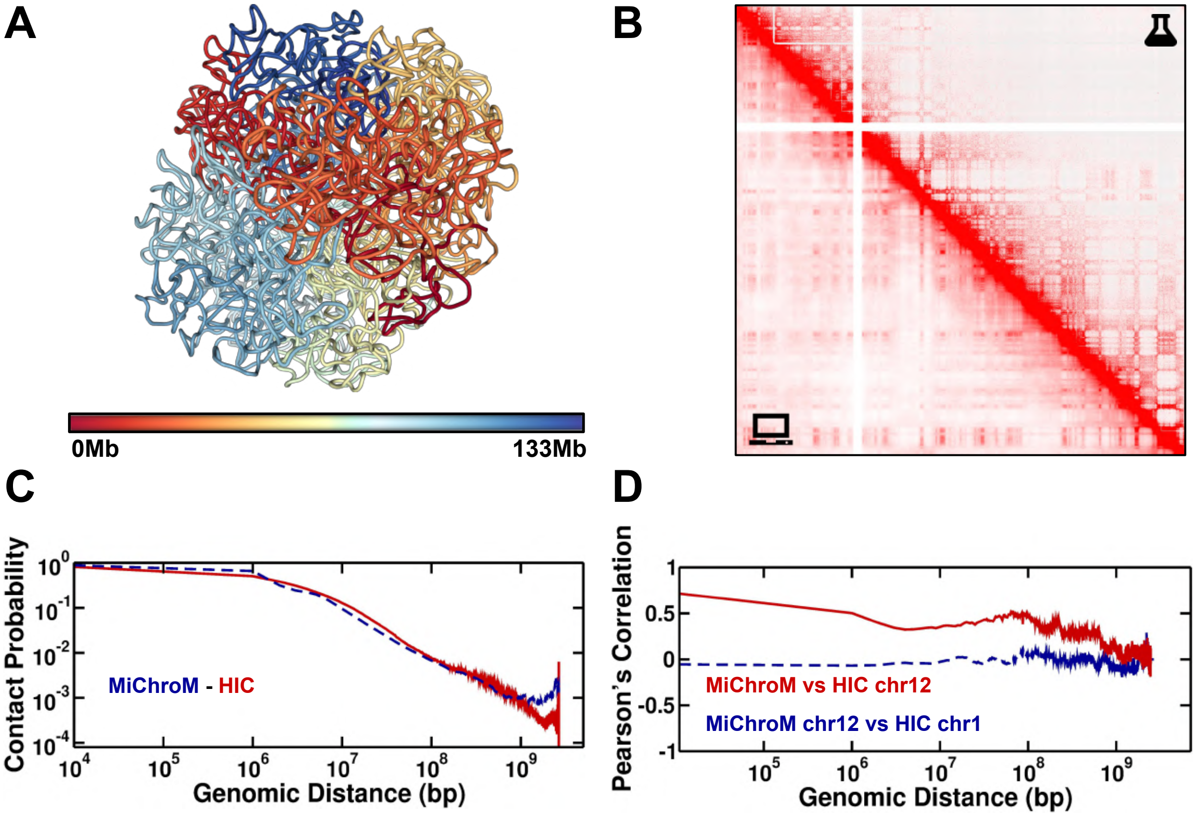

Figure S12: The structural ensemble of chromosome 12, cell line A549, generated by simulations using the MiChroM+MEGABASE pipeline. A - Three-dimensional representative structure colored by index, red to blue. B - Comparison between Hi-C maps obtained from wet-lab experiments (top) and *in silico* (bottom). The Hi-C maps predicted from simulations have been sampled from an ensemble of 500 thousand structures. C - Contact probability as a function of the genomic distance. The solid red line is the curve extracted from the experimental data. The dashed blue line is the data obtained from the *in silico* HiC. D - Pearson's correlation between experimental and simulated Hi-C maps of chromosome 12 as a function of the genomic distance, shown by the solid red line. As a term of comparison the dashed blue line shows the correlation between Hi-C maps of different chromosomes.

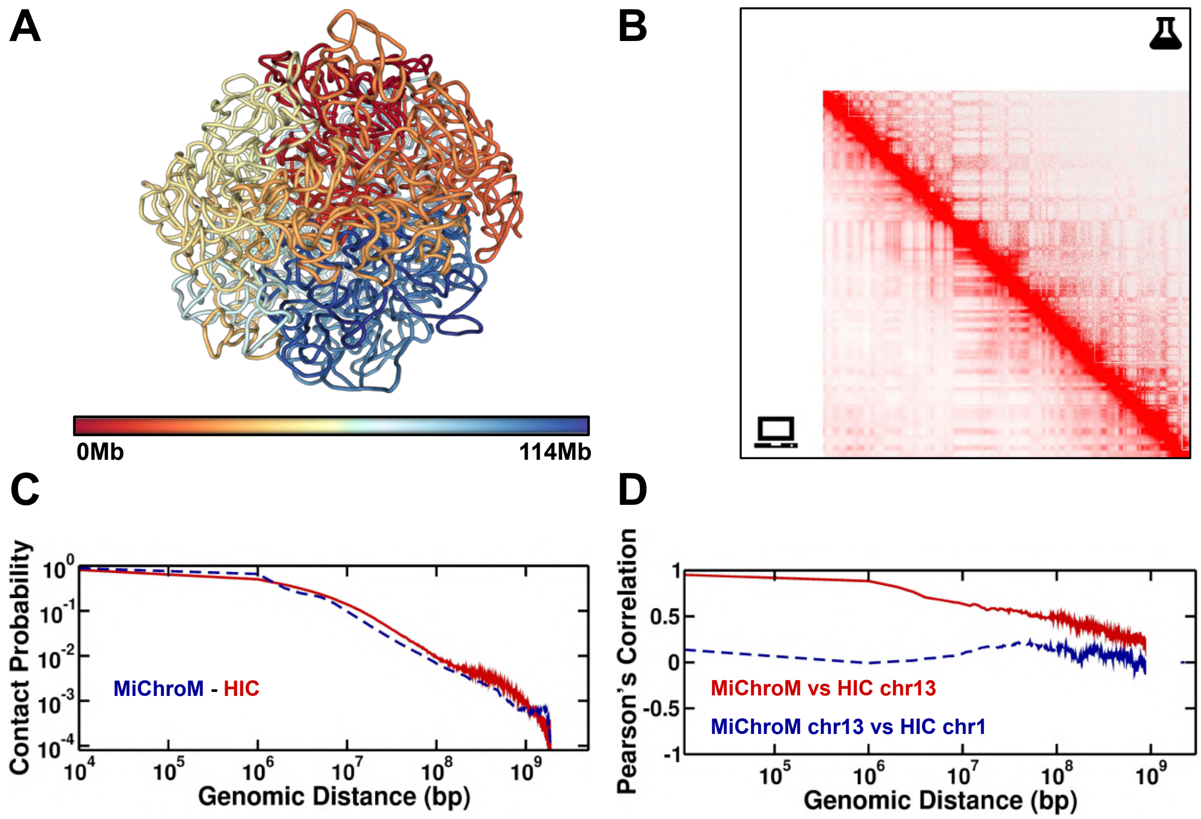

Figure S13: The structural ensemble of chromosome 13, cell line A549, generated by simulations using the MiChroM+MEGABASE pipeline. A - Three-dimensional representative structure colored by index, red to blue. B - Comparison between Hi-C maps obtained from wet-lab experiments (top) and *in silico* (bottom). The Hi-C maps predicted from simulations have been sampled from an ensemble of 500 thousand structures. C - Contact probability as a function of the genomic distance. The solid red line is the curve extracted from the experimental data. The dashed blue line is the data obtained from the *in silico* HiC. D - Pearson's correlation between experimental and simulated Hi-C maps of chromosome 13 as a function of the genomic distance, shown by the solid red line. As a term of comparison the dashed blue line shows the correlation between Hi-C maps of different chromosomes.

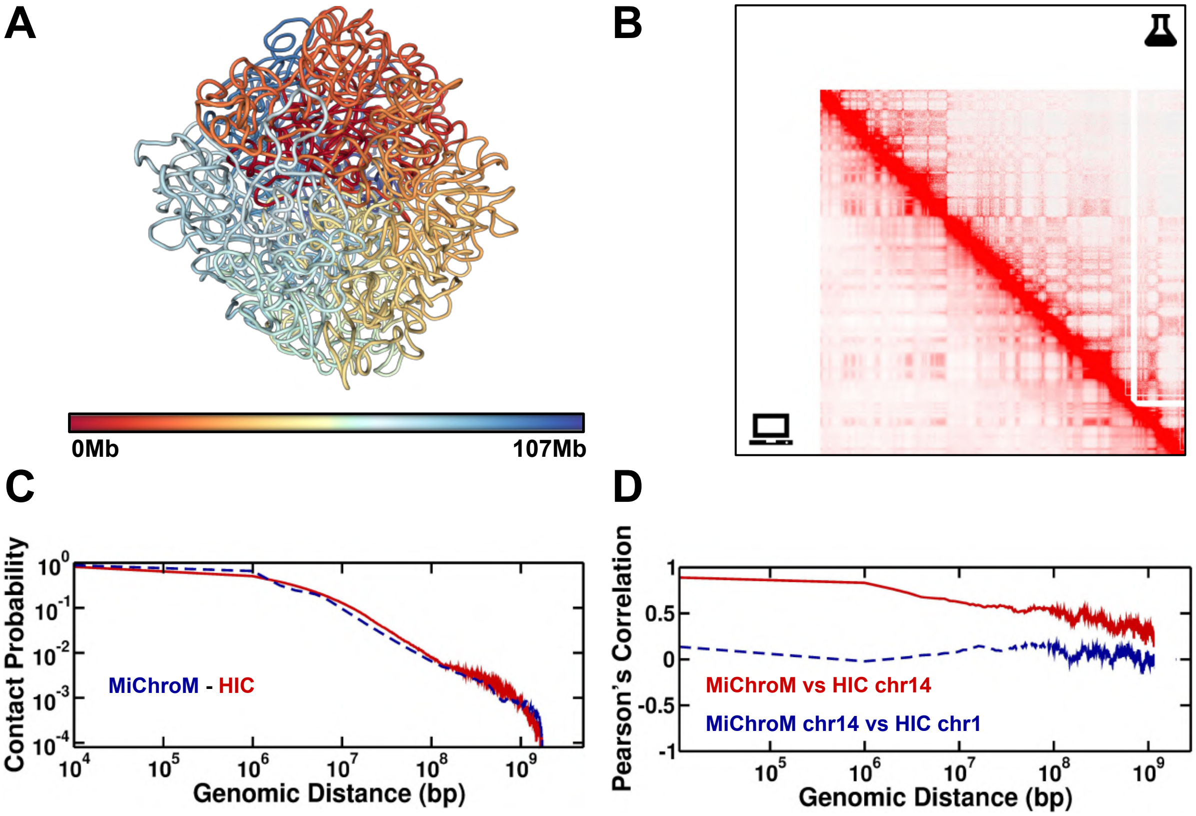

Figure S14: The structural ensemble of chromosome 14, cell line A549, generated by simulations using the MiChroM+MEGABASE pipeline. A - Three-dimensional representative structure colored by index, red to blue. B - Comparison between Hi-C maps obtained from wet-lab experiments (top) and *in silico* (bottom). The Hi-C maps predicted from simulations have been sampled from an ensemble of 500 thousand structures. C - Contact probability as a function of the genomic distance. The solid red line is the curve extracted from the experimental data. The dashed blue line is the data obtained from the *in silico* HiC. D - Pearson's correlation between experimental and simulated Hi-C maps of chromosome 14 as a function of the genomic distance, shown by the solid red line. As a term of comparison the dashed blue line shows the correlation between Hi-C maps of different chromosomes.

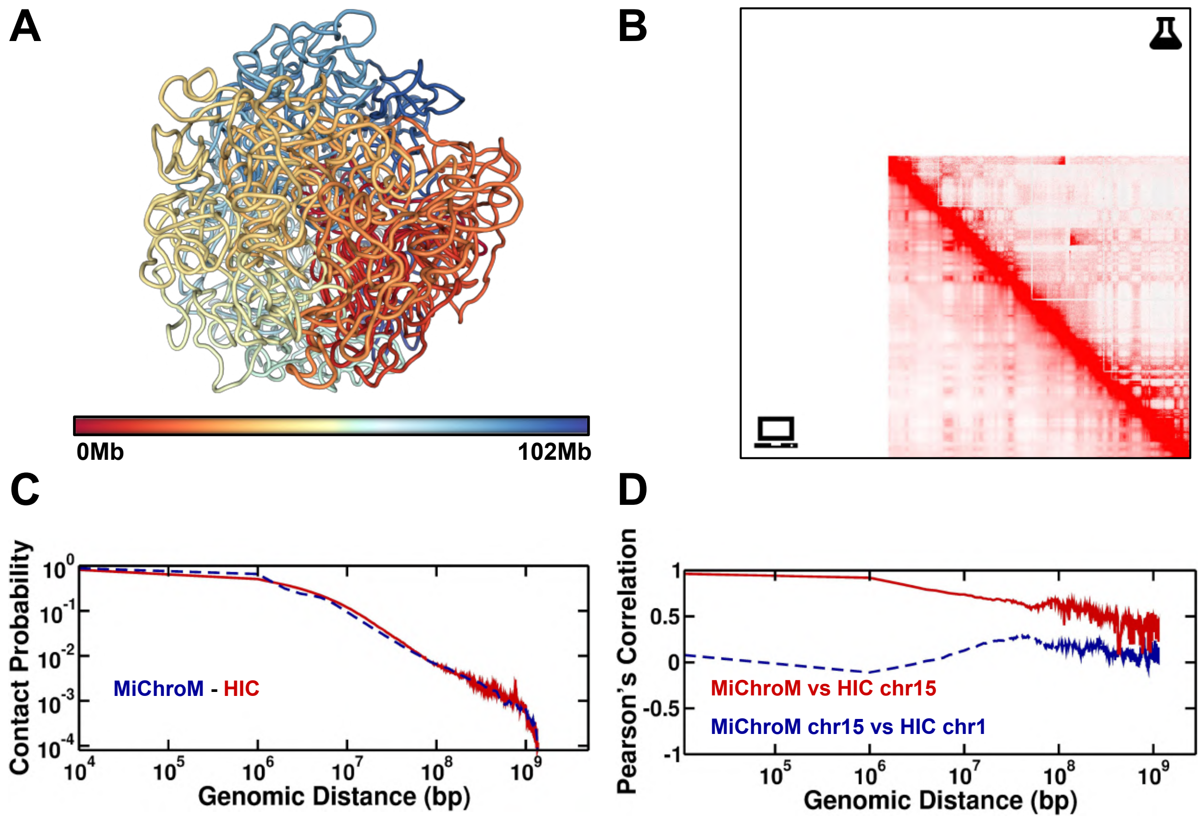

Figure S15: The structural ensemble of chromosome 15, cell line A549, generated by simulations using the MiChroM+MEGABASE pipeline. A - Three-dimensional representative structure colored by index, red to blue. B - Comparison between Hi-C maps obtained from wet-lab experiments (top) and *in silico* (bottom). The Hi-C maps predicted from simulations have been sampled from an ensemble of 500 thousand structures. C - Contact probability as a function of the genomic distance. The solid red line is the curve extracted from the experimental data. The dashed blue line is the data obtained from the *in silico* HiC. D - Pearson's correlation between experimental and simulated Hi-C maps of chromosome 15 as a function of the genomic distance, shown by the solid red line. As a term of comparison the dashed blue line shows the correlation between Hi-C maps of different chromosomes.

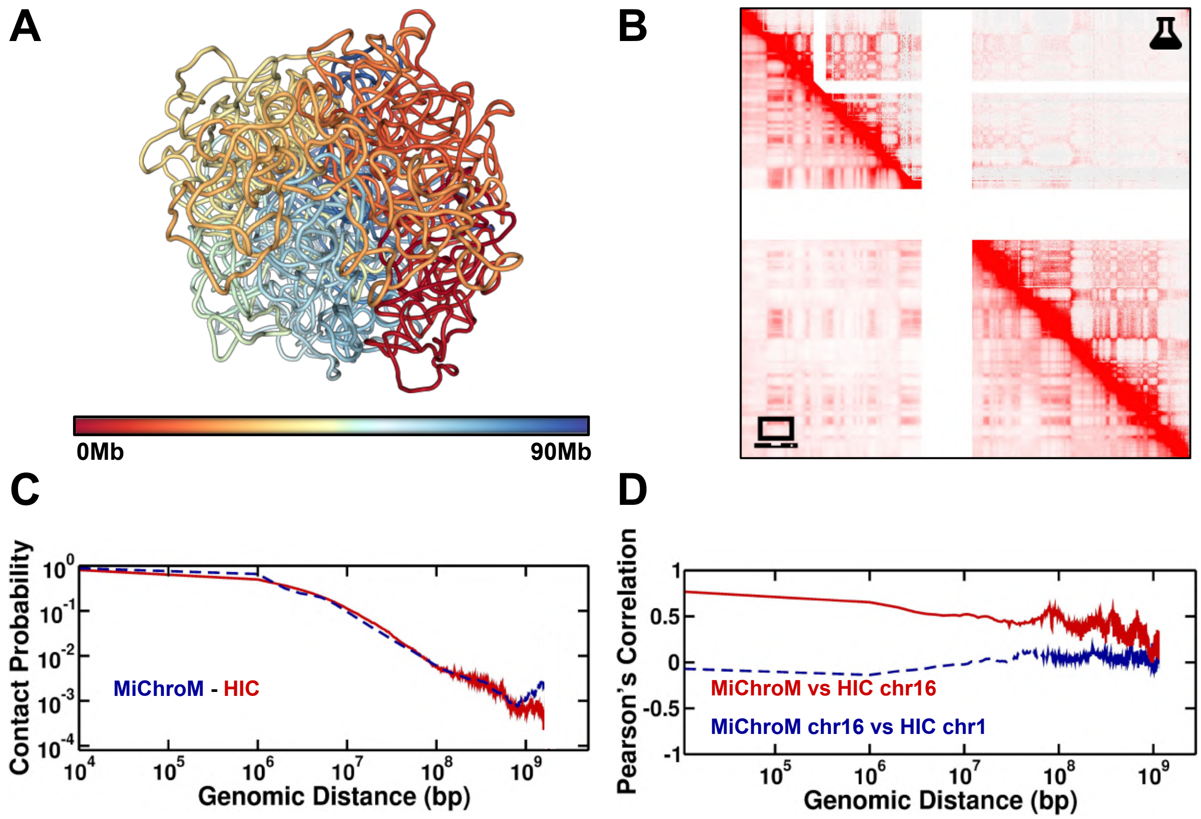

Figure S16: The structural ensemble of chromosome 16, cell line A549, generated by simulations using the MiChroM+MEGABASE pipeline. A - Three-dimensional representative structure colored by index, red to blue. B - Comparison between Hi-C maps obtained from wet-lab experiments (top) and *in silico* (bottom). The Hi-C maps predicted from simulations have been sampled from an ensemble of 500 thousand structures. C - Contact probability as a function of the genomic distance. The solid red line is the curve extracted from the experimental data. The dashed blue line is the data obtained from the *in silico* HiC. D - Pearson's correlation between experimental and simulated Hi-C maps of chromosome 16 as a function of the genomic distance, shown by the solid red line. As a term of comparison the dashed blue line shows the correlation between Hi-C maps of different chromosomes.

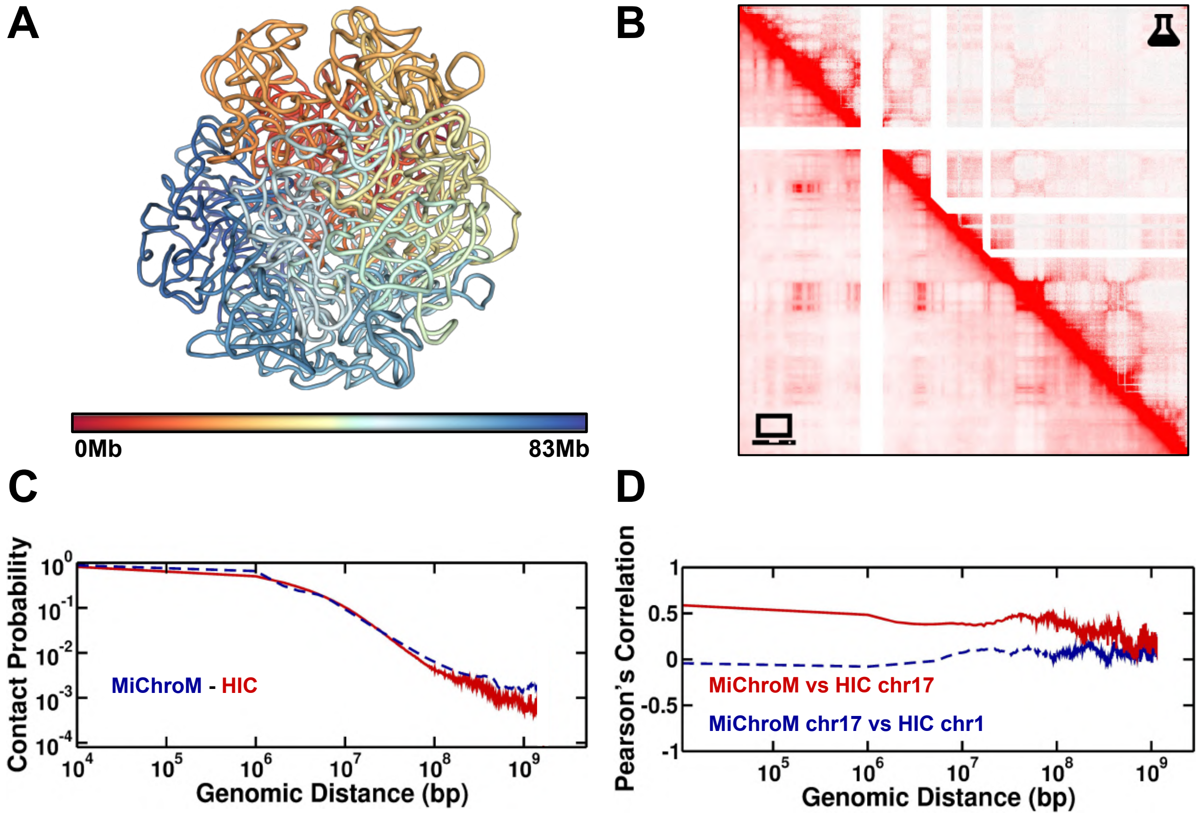

Figure S17: The structural ensemble of chromosome 17, cell line A549, generated by simulations using the MiChroM+MEGABASE pipeline. A - Three-dimensional representative structure colored by index, red to blue. B - Comparison between Hi-C maps obtained from wet-lab experiments (top) and *in silico* (bottom). The Hi-C maps predicted from simulations have been sampled from an ensemble of 500 thousand structures. C - Contact probability as a function of the genomic distance. The solid red line is the curve extracted from the experimental data. The dashed blue line is the data obtained from the *in silico* HiC. D - Pearson's correlation between experimental and simulated Hi-C maps of chromosome 17 as a function of the genomic distance, shown by the solid red line. As a term of comparison the dashed blue line shows the correlation between Hi-C maps of different chromosomes.

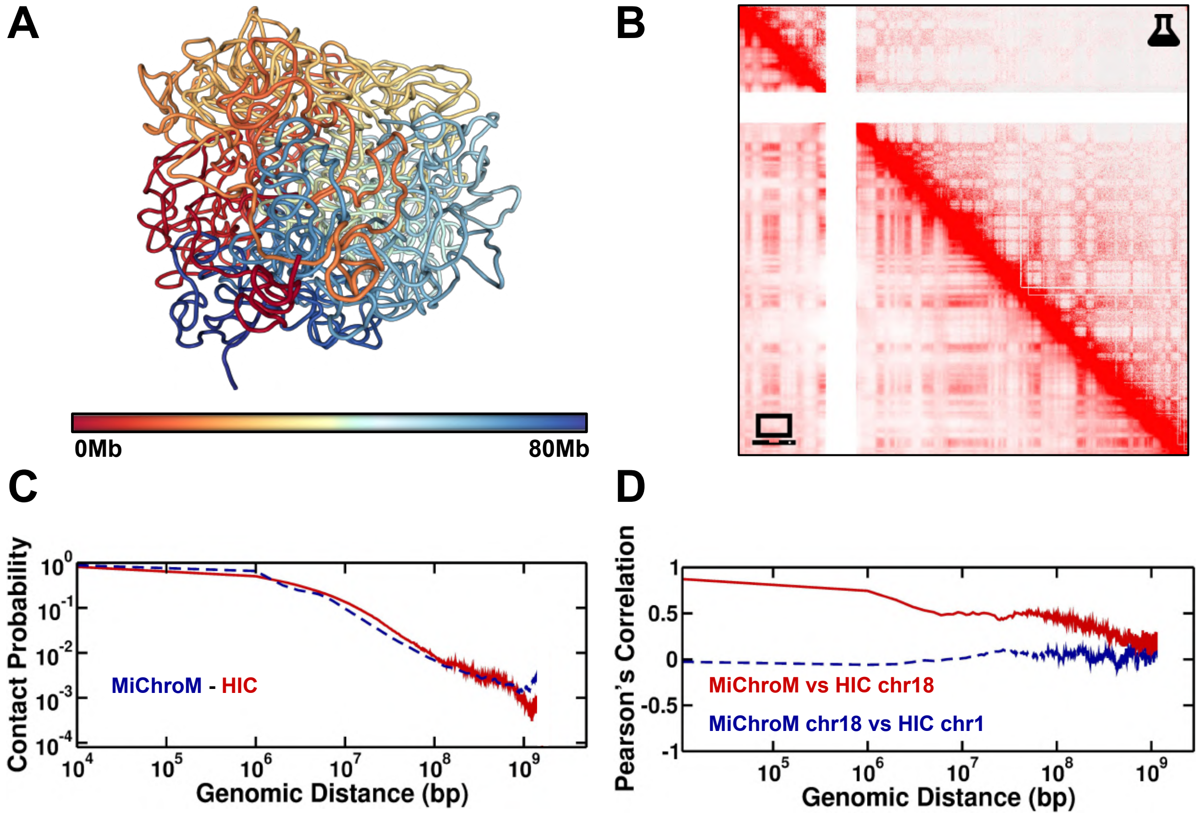

Figure S18: The structural ensemble of chromosome 18, cell line A549, generated by simulations using the MiChroM+MEGABASE pipeline. A - Three-dimensional representative structure colored by index, red to blue. B - Comparison between Hi-C maps obtained from wet-lab experiments (top) and *in silico* (bottom). The Hi-C maps predicted from simulations have been sampled from an ensemble of 500 thousand structures. C - Contact probability as a function of the genomic distance. The solid red line is the curve extracted from the experimental data. The dashed blue line is the data obtained from the *in silico* HiC. D - Pearson's correlation between experimental and simulated Hi-C maps of chromosome 18 as a function of the genomic distance, shown by the solid red line. As a term of comparison the dashed blue line shows the correlation between Hi-C maps of different chromosomes.

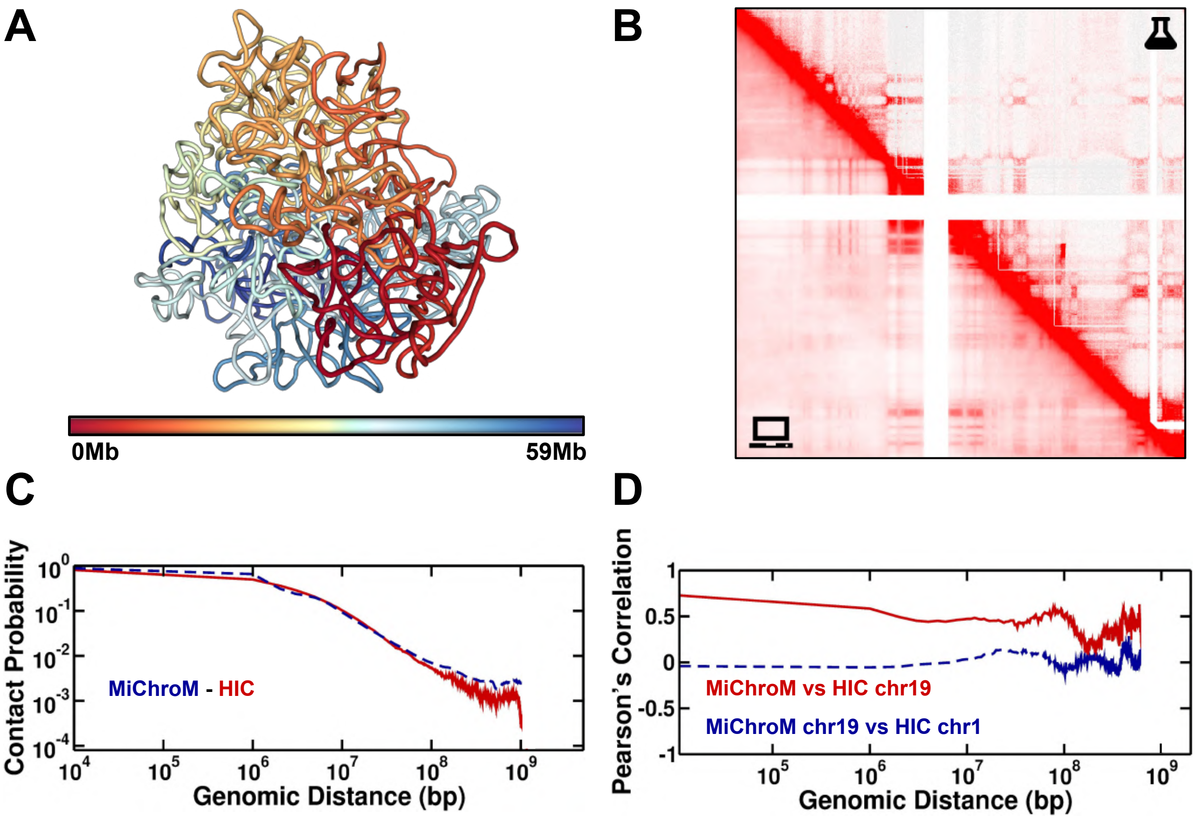

Figure S19: The structural ensemble of chromosome 19, cell line A549, generated by simulations using the MiChroM+MEGABASE pipeline. A - Three-dimensional representative structure colored by index, red to blue. B - Comparison between Hi-C maps obtained from wet-lab experiments (top) and *in silico* (bottom). The Hi-C maps predicted from simulations have been sampled from an ensemble of 500 thousand structures. C - Contact probability as a function of the genomic distance. The solid red line is the curve extracted from the experimental data. The dashed blue line is the data obtained from the *in silico* HiC. D - Pearson's correlation between experimental and simulated Hi-C maps of chromosome 19 as a function of the genomic distance, shown by the solid red line. As a term of comparison the dashed blue line shows the correlation between Hi-C maps of different chromosomes.

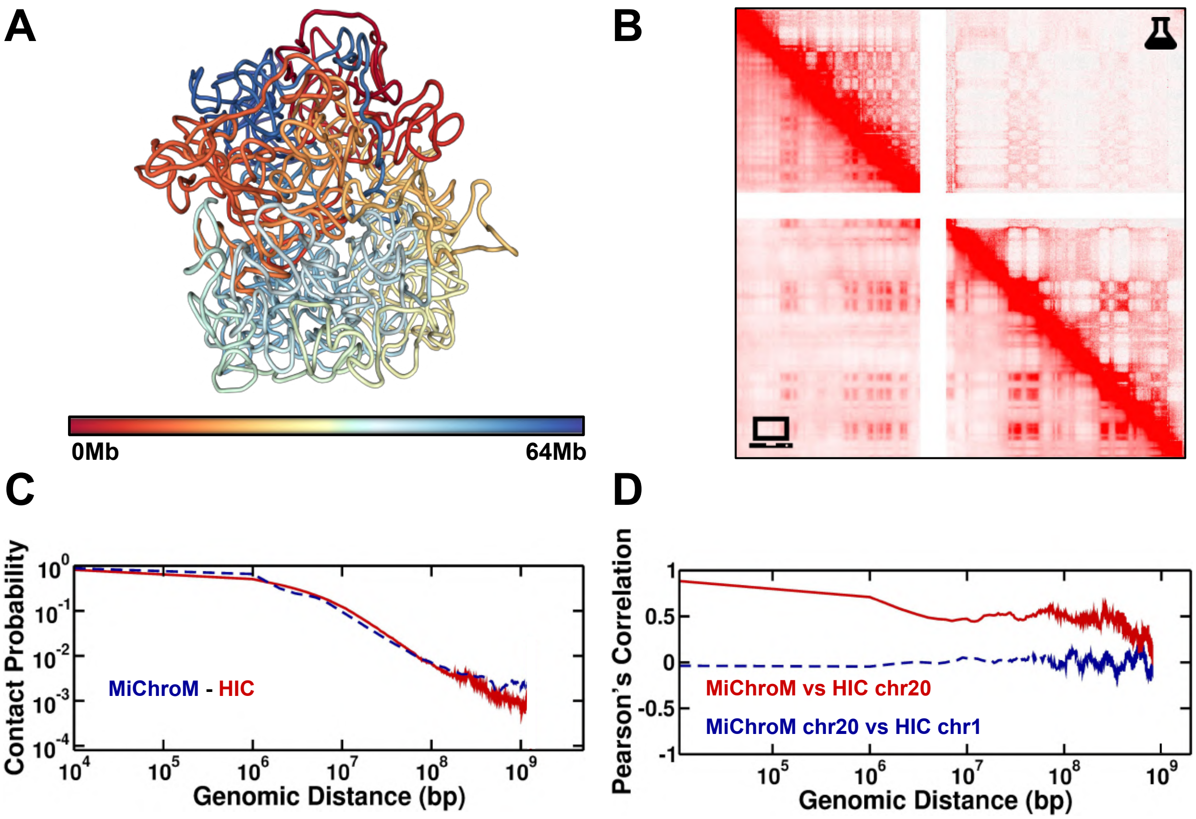

Figure S20: The structural ensemble of chromosome 20, cell line A549, generated by simulations using the MiChroM+MEGABASE pipeline. A - Three-dimensional representative structure colored by index, red to blue. B - Comparison between Hi-C maps obtained from wet-lab experiments (top) and *in silico* (bottom). The Hi-C maps predicted from simulations have been sampled from an ensemble of 500 thousand structures. C - Contact probability as a function of the genomic distance. The solid red line is the curve extracted from the experimental data. The dashed blue line is the data obtained from the *in silico* HiC. D - Pearson's correlation between experimental and simulated Hi-C maps of chromosome 20 as a function of the genomic distance, shown by the solid red line. As a term of comparison the dashed blue line shows the correlation between Hi-C maps of different chromosomes.

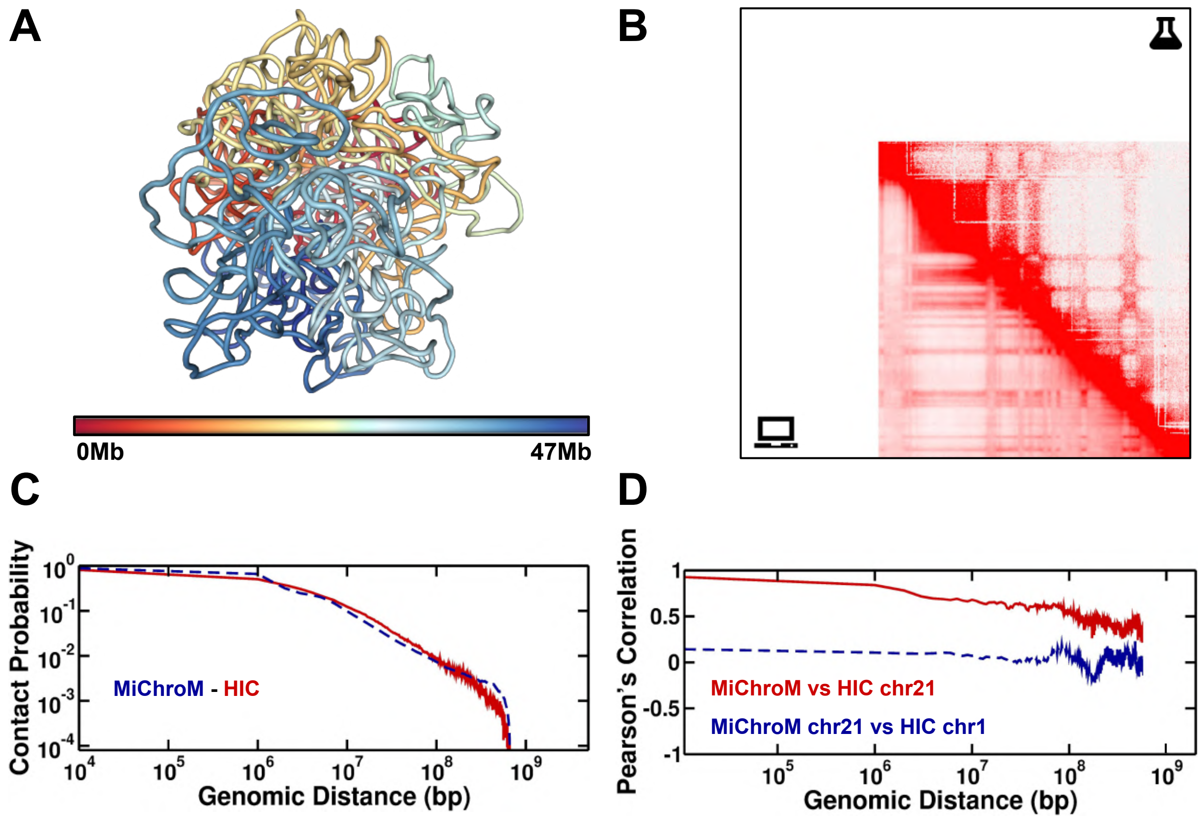

Figure S21: The structural ensemble of chromosome 21, cell line A549, generated by simulations using the MiChroM+MEGABASE pipeline. A - Three-dimensional representative structure colored by index, red to blue. B - Comparison between Hi-C maps obtained from wet-lab experiments (top) and *in silico* (bottom). The Hi-C maps predicted from simulations have been sampled from an ensemble of 500 thousand structures. C - Contact probability as a function of the genomic distance. The solid red line is the curve extracted from the experimental data. The dashed blue line is the data obtained from the *in silico* HiC. D - Pearson's correlation between experimental and simulated Hi-C maps of chromosome 21 as a function of the genomic distance, shown by the solid red line. As a term of comparison the dashed blue line shows the correlation between Hi-C maps of different chromosomes.

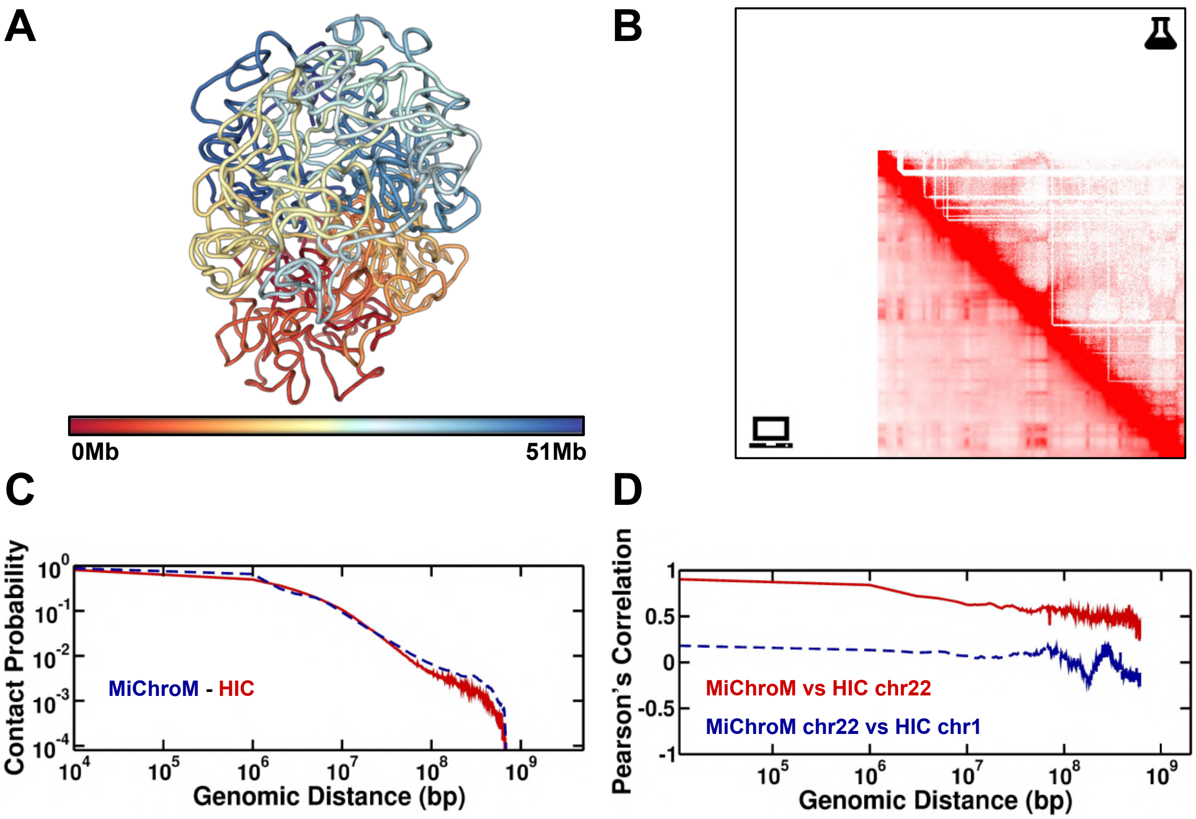

Figure S22: The structural ensemble of chromosome 22, cell line A549, generated by simulations using the MiChroM+MEGABASE pipeline. A - Three-dimensional representative structure colored by index, red to blue. B - Comparison between Hi-C maps obtained from wet-lab experiments (top) and *in silico* (bottom). The Hi-C maps predicted from simulations have been sampled from an ensemble of 500 thousand structures. C - Contact probability as a function of the genomic distance. The solid red line is the curve extracted from the experimental data. The dashed blue line is the data obtained from the *in silico* HiC. D - Pearson's correlation between experimental and simulated Hi-C maps of chromosome 22 as a function of the genomic distance, shown by the solid red line. As a term of comparison the dashed blue line shows the correlation between Hi-C maps of different chromosomes.
